# A chromosome-level genome of the helmet catfish (*Cranoglanis bouderius*)

**DOI:** 10.3389/fgene.2022.962406

**Published:** 2022-08-10

**Authors:** Yuan Xu, Feng Shao, Weitao Chen, Luyun Ni, Zuogang Peng

**Affiliations:** ^1^ Key Laboratory of Freshwater Fish Reproduction and Development (Ministry of Education), Southwest University School of Life Sciences, Chongqing, China; ^2^ Pearl River Fisheries Research Institute, Chinese Academy of Fishery Sciences, Guangzhou, China; ^3^ Academy of Plateau Science and Sustainability, Qinghai Normal University, Xining, China

**Keywords:** helmet catfish, chromosome-level assembly, comparative genomics, Hi-C, HIFI

## Introduction

It is said that the history of life has been written in the genome ( Blaxter et al., 2022 ), and indeed, many significant questions pertaining to the evolution and ecology of life on earth will only be addressable when whole-genome data representing divergences at all branches in the tree of life are available. Genomes will serve as core databanks for inferring the phylogeny of all life and contribute to gaining a comprehensive understanding of species biology (Blaxter et al., 2022). Consequently, accruing genomic resources of important species in tree of life has significant implications for evolutionary studies and other fields of biological research.

The fish family Cranoglanididae (Teleostei: Siluriformes) is notable in that it comprises a single genus containing just three species (Fricke et al., 2022), one of which is the helmet catfish (*Cranoglanis bouderius*), an economically important aquaculture fish endemic to China. They are only distributed in the Pearl River Basin in South China and mainly live on shrimps and small fishes. With respect to commercial value, this species has a number of favorable traits, including a high proportion of edible flesh, high nutritional value, strong disease resistance, and fewer intermuscular spines ( Wu et al., 2010 ). However, the wild resources of this fish have declined markedly in recent years, as a consequence of overfishing, and in 2011 it was placed on the IUCN Red List of Threatened Species (www.iucnredlist.org). Previous studies on *C. bouderius* have focused on morphology ( Diogo et al., 2002; Luckenbill and Lundberg, 2009 ), physiology ( Zhou et al., 2003; Chen et al., 2016 ), genetic diversity ( Cheng et al., 2009 ), and molecular characterization of the complete mitochondrial genome ( Peng et al., 2006 ). However, despite this extensive research, the lack of a high-quality chromosome-level reference genome for *C. bouderius* has significantly hindered in-depth research on the evolution, breeding, and conservation of this fish.

To rectify this deficiency, in this study, using Illumina, Nanopore, PacBio, and Hi-C technologies, we constructed the first chromosome-level genome for *C. bouderius*, thereby providing an available genomic resource for future studies on the conservation, artificial breeding, and evolution of this fish.

### Data description

All Illumina, Nanopore, and PacBio sequencing data generated in this study are presented in Supplementary Table S1. Estimates of genome size and heterozygosity were performed using a total of 36.82 Gb of filtered Illumina sequencing data based on 17-mer analysis. The main peak was located at a depth of 34 with a predicted genome size of 931.11 Mb and an estimated percentage heterozygosity of 0.5% (Supplementary Table S2; Supplementary Figure S1).

For *de novo* assembly of the *C. bouderius* genome, we used 92.50 Gb of ONT long reads generated using Oxford Nanopore Technologies to construct a preliminary assembly, and subsequently used 13.62 Gb of PacBio HiFi reads and 36.82 Gb of Illumina short reads to polish the preliminary assembly, yielding 881.33 Mb of genomic DNA sequences, comprising 231 contigs with a contig N50 length of 20.01 Mb (Supplementary Table S3). RNA-seq short reads from different tissues were aligned to the assembly genome using HISAT2, with the percentage of aligned reads ranging from 92.02 to 93.98% (Supplementary Table S4). Completeness of the *C. bouderius* genome was assessed based on BUSCO analysis using the Actinopterygii_odb10 database, with 95.71% (3,484) of BUSCO genes being identified in the *C. bouderius* genome (Supplementary Table S5), thereby indicating the high level of completeness of the genome assembly.

**FIGURE 1:**
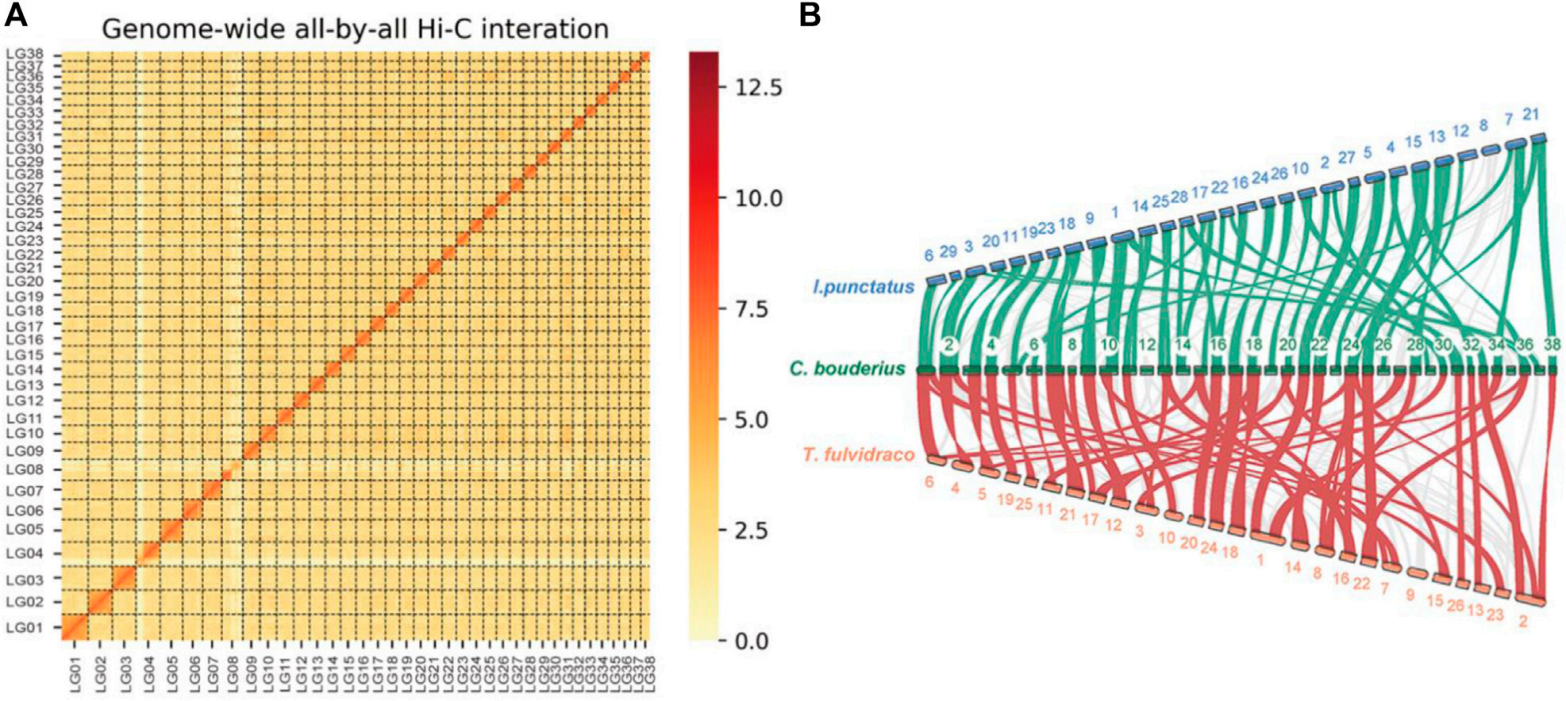
Characteristics of the *Cranoglanis bouderius* genome. **(A)** A Hi-C contact map of the *C. bouderius* genome. LGs 1–38: *Lachesis* groups 1 to 38, representing the 38 chromosomes of *C. bouderius*. **(B)** Gene collinearity between *C. bouderius* and the two related fish species *Tachysurus fulvidraco* and *Ictalurus punctatus*. The green and red links denote block scores exceeding 200 between *I. punctatus* and *C. bouderius*, and between *C. bouderius* and *T. fulvidraco*, respectively. *C. bouderius* chromosomes are numbered in ascending order.

A total of 138.25 Gb of clean reads obtained from the Hi-C library were used for chromosome-level genome assembly. We constructed a high-quality chromosome-level *C. bouderius* genome of 876.63 Mb, with 214 contigs being successfully anchored and orientated on 38 chromosomes, ranging in size from 13.73 to 39.43 Mb (Supplementary Table S6). Among them, 10 chromosomes were gapless, such as LG07, LG15, and LG17 (Supplementary Table S6). A genome-wide Hi-C interaction heatmap indicated that the interaction signal strength around the diagonal was significantly stronger than that at other position within each chromosome (Figure 1A). To the best of our knowledge, *C. bouderius* has the highest number of chromosomes among all Siluriformes for which chromosome-level genomes have been published. Comparative gene collinearity analysis indicated that despite interspecific differences in chromosome numbers, most chromosomes showed good collinearity (Figure 1B), thereby further verifying the reliability of the genomic data generated in this study.

The *C. bouderius* genome was found to comprise 49.55% repetitive sequences, with transposons accounting for 43.87% of the assembled genome (Supplementary Table S7), among which, the largest proportion (29.2%) consists of DNA transposons, followed by LTRs (7.29%), LINEs (6.32%), and SINEs (1.07%). On the basis of a combination of homologous, *de novo*, and RNA-seq prediction approaches, we annotated 21,715 protein-coding genes, with average gene, coding sequence, and exon and intron lengths of 20,496.52, 1,761.49, 167.97, and 1,974.86 bp, respectively, which are consistent with the distribution trends of these parameters in the released genomes used for annotation (Supplementary Figure S2). To assess the completeness of genome annotation we also applied BUSCO in conjunction with Actinopterygii_odb10 database, with the results indicating that 96.07% of the conserved single-copy genes in the *C. bouderius* genome were completely predicted (Supplementary Table S8). In addition, non-coding RNA annotation revealed that the genome contains a total of 1,950 miRNAs, 5,792 tRNAs, and 795 rRNAs, whereas with respect to functional annotations, we succeeded in annotating 21,085 genes using at least one of the referenced databases (KEGG, KOG, NR, SwissProt, and GO), accounting for 97.10% of the predicted protein-coding genes (Supplementary Table S9).

**FIGURE 2:**
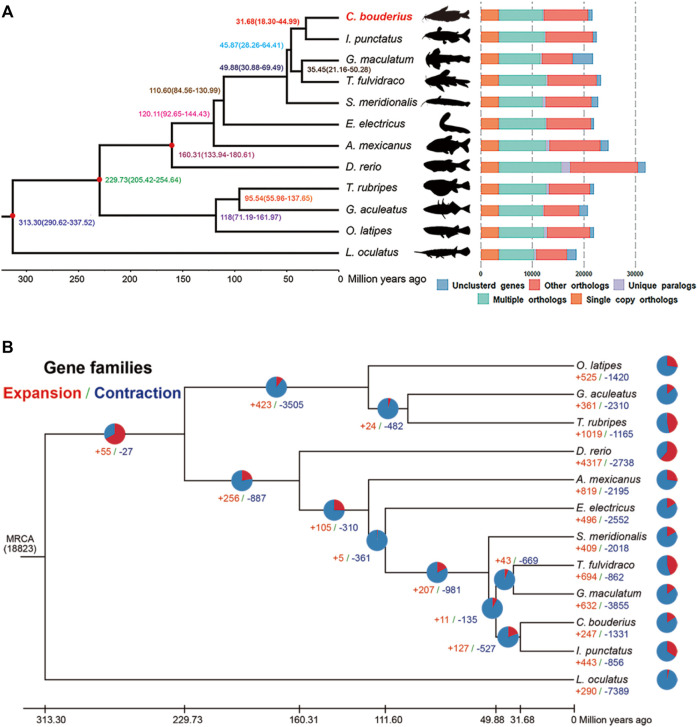
Phylogenetic and evolutionary analyses of *Cranoglanis bouderius* and other teleost fishes. **(A)** Divergence time estimation and gene clustering in *C. bouderius* and other fish species. The phylogenetic tree of 12 species was constructed using 3436 single-copy orthologous genes. The numbers on the branches denote estimated divergence times in millions of years ago and the three red nodes represent fossil calibration points used for divergence time estimation. **(B)** The expansion and contraction of gene families in 12 fish species genomes. The pie charts and numbers represent the proportion and specific values of expanded (red) and contracted (blue) gene families, respectively. MRCA: most recent common ancestor.

The phylogenetic relationships between *C. bouderius* and other vertebrates, and the respective divergence times were deduced based on an analysis of 3,436 single-copy orthologous genes from the genomes of 12 teleost fishes (Figure 2A). Our findings indicated that *C. bouderius* is most closely related to species within the family Ictaluridae, which is consistent with the assessments of previously published phylogenetic studies ( Sullivan et al., 2006 ). The five species of Siluriformes were found to form a monophyletic group, and Siluriformes and Gymnotiformes were identified as sister groups. In addition, we estimated that *C. bouderius* diverged from *Ictalurus punctatus* approximately 31.68 million years ago (Mya), whereas the time of the divergence between Siluriformes and Gymnotiformes was around 110.60 Mya.

It is speculated that the expansion and contraction of gene families may play important roles in environmental adaptation, and on the basis of gene family analysis, we identified 247 and 1,331 gene families that have undergone expansion and contraction, respectively, since the aforementioned divergence from *I. punctatus* (Figure 2B). Among these gene families, 45 and 38 families were identified as being significantly expanded and contracted, respectively (Supplementary Table S10). Further enrichment analysis revealed that the expanded genes were enriched in 21 KEGG pathways (*p* < 0.05) (Supplementary Table S11), most of which are associated with olfaction, immunity, and nutrient metabolism. These findings accordingly provide a new perspective for further studies on growth, metabolism, and adaptation to a benthic habitat. Similarly, contracted genes were identified as being enriched in 18 KEGG pathways (*p* < 0.05) (Supplementary Table S12), the most highly represented of which were “Complement and coagulation cascades,” “Tight junction,” and “Pathogenic *Escherichia coli* infection.”

## Materials and methods

### Ethics statement

All animal experiments conducted in this study were reviewed and approved by the Ethics Committee of Southwest University (NO.20200715-01).

### Sampling, library construction, and sequencing

For the purposes of genome and transcriptome sequencing, we used samples obtained from a female *C. bouderius* (Supplementary Figure S3) captured from the main channel of the Pearl River in Guangdong Province, China (23°8′27″N, 111°45′35″E). Blood samples were collected for DNA extraction performed using a QIAGEN^®^ Genomic kit (Qiagen, Hilden, Germany). A subsequently constructed DNA library (200–400 bp) was sequenced using the MGISEQ 2000 platform (Korostin et al., 2020). Low-quality reads in raw data were filtered out using fastp ( Chen et al., 2018 ) (default parameters). An ONT library was prepared from the long DNA fragments selected using the BluePippin system (Sage Science, United States), with subsequent sequencing using a PromethION sequencer (Oxford Nanopore Technologies, United Kingdom). The FAST5 files generated using a Nanopore sequencer were converted to FASTQ format using Guppy ( Wick et al., 2019 ), and the raw data with mean_qsocre_template values <7 were filtered. Using the PacBio protocol (Pacific Biosciences, CA, United States), we constructed a SMRTbell library by shearing genomic DNA with g-TUBE (Covaris, United States). Target size libraries (10–20 kb) were sequenced using a PacBio Sequal II sequencer to generate HiFi reads.

RNA from 11 tissue types of the aforementioned fish (adipose fin, brain, fins, gills, gonad, heart, head kidney, kidney, liver, muscle, and spleen) was extracted using an RNeasy Plus Mini Kit (Qiagen), and validated RNA samples were used for complementary DNA library construction using a TruSeq Sample Preparation Kit (Illumina) and Illumina HiSeq4000 sequencer. Equal amounts of qualified RNA from each of the 11 tissue types were pooled and reverse-transcribed using an SQK-PCS109 sequencing kit (Oxford Nanopore Technologies) for ONT libraries preparation and sequencing (Nanopore PromethION). To prevent the potential influence of adapter sequences and low-quality reads in subsequent analyses, the raw reads were filtered as described for DNA.

### Genome features based on K-Mer analysis and genome assembly

Clean reads of sequences obtained using the MGISEQ 2000 platform were used for estimation of genome size and heterozygosity based on 17-mer frequency distribution analysis using jellyfish software ( Marcais and Kingsford, 2011 ).

Long reads generated using the Nanopore platform were used for NextDenovo-based *de novo* assembly (reads_cutoff:1k, seed_cutoff:20k, https://github.com/Nextomics/NextDenovo). To enhance the accuracy of assembly, polishing of the short and long reads was performed using Racon (https://github.com/isovic/racon) and Nextpolish (https://github.com/Nextomics/NextPolish), respectively, with default parameters.

### Chromosome-level genome assembly using Hi-C and assessment

For chromosome-level assembly of the *C. bouderius* genome**,** genomic DNA was extracted from kidney samples for Hi-C library construction, and sequencing was performed using the MGISEQ 2000 platform. The Hi-C data thus obtained was initially filtered using fastp (default parameters), and the clean paired-end reads were then mapped to the primary genome using Bowtie2 ( Langmead and Salzberg, 2012 ) to generate unique mapped paired-end reads. Valid interacting paired reads were obtained using HiC-Pro from uniquely mapped paired-end reads. A chromosome-level assembly was generated using LACHESIS ( Burton et al., 2013 ) with the following parameters: CLUSTER MIN RE SITES = 100; CLUSTER MAX LINK DENSITY = 2.5; CLUSTER NONINFORMATIVE RATIO = 1.4; ORDER MIN N RES TRUNK = 60; ORDER MIN N RES SHREDS = 60. A genome-wide Hi-C heat map was constructed based on the interaction between different scaffolds.

We assessed the completeness of the assembly using two strategies, namely, BUSCO ( Simao et al., 2015 ) and RNA-seq read mapping. Core genes of the Actinopterygii database were searched against the *C. bouderius* genome using BUSCO, and RNA-seq reads were mapped to the genome using HISAT2 ( Kim et al., 2015 ) with default parameters. Furthermore, to access the accuracy of the scaffolding results, we performed gene collinearity analysis between *C. bouderius* and two related species, *I. punctatus* and *Tachysurus fulvidraco*, using MCScanX ( Wang et al., 2012 ).

### Repeat sequence annotation

Tandem repeats were annotated using GMATA ( Wang and Wang, 2016 ) and TRF ( Benson, 1999 ) with default parameters. With respect to the annotation of transposable elements, we applied two strategies, namely, *de novo* and homology-based prediction, using. MITE-hunter ( Han and Wessler, 2010 ), LTR_finder ( Xu and Wang, 2007 ), and RepeatModeler ( Bedell et al., 2000 ) with default parameters for *de novo* prediction. For homology-based prediction, we initially merged the ab initio library and Repbase ( Bao et al., 2015 ) TE library as a final repeat sequence library, and the aligned this to the genome using RepeatMasker ( Tarailo-Graovac and Chen, 2009 ). The outcomes of the two predictions were integrated as the final non-redundant repeat sequence annotation.

### Gene prediction and functional annotation

Three independent methods, namely, homologous, *de novo*, and RNA-seq prediction, were used for gene prediction*.* For homology-based prediction, protein sequences from the genomes of the following nine vertebrates were aligned to the *C. bouderius* genome using GeMoMa ( Keilwagen et al., 2016 ): channel catfish (*I. punctatus*), Chinese yellow catfish (*T. fulvidraco*), giant devil catfish (*Bagarius yarrelli*), humans (*Homo sapiens*), Japanese puffer (*Takifugu rubripes*), medaka (*Ozyzias latipes*), red-tail catfish (*Hemibagrus wyckioides*), Tibetan catfish (*Glyptosternon maculatum*), and zebrafish (*Danio rerio*). For *de novo* prediction, we used AUGUSTUS ( Stanke et al., 2008 ) with default parameters and a training set generated from RNA-seq reads assembled using StringTie ( Pertea et al., 2015 ) and PASA ( Haas et al., 2003 ). For transcriptome-based prediction, the transcripts assembled based on full-length and short reads were merged with open reading frames and predicted using PASA. The final integrated gene set was produced using EVidenceModeler ( Haas et al., 2008 ), with sequences containing transposons being removed using TransposonPSI (http://transposonpsi.sourceforge.net/). The accuracy of gene prediction was assessed based on BUSCO analysis using the Actinopterygii_odb9 database, and the NR ( Marchler-Bauer et al., 2011 ), KOG ( Tatusov et al., 2003 ), KEGG ( Kanehisa et al., 2014 ), and SwissProt ( Bateman et al., 2021 ) databases were used to functionally annotate these predicted genes *via* BLASTP searches with an E-value cutoff of 1e-5. In addition, Gene Ontology (GO) annotation was performed using InterProScan ( Zdobnov and Apweiler, 2001 ) with default parameters.

For the annotation of non-coding RNAs, we used tRNAscan-SE ( Lowe and Eddy, 1997 ) and RNAmmer ( Lagesen et al., 2007 ) to annotate tRNAs and rRNAs, respectively, whereas microRNAs and small nuclear RNAs were respectively predicted using INFERNAL ( Nawrocki and Eddy, 2013 ) and Rfam database ( Daub et al., 2015 ).

### Comparative genomic analyses

For the purpose of identifying orthologous gene families using OrthoMCL ( Li et al., 2003 ), in addition to the protein sequences of *C. bouderius,* we downloaded those for the following 11 species from the NCBI and GIGADB databases: channel catfish, Chinese yellow catfish, electric eel (*Electrophorus electricus*), Japanese puffer, medaka, Mexican tetra (*Astyanax mexicanus*), southern catfish (*Silurus meridionalis*), spotted gar (*Lepisosteus oculatus*), three-spined stickleback (*Gasterosteus aculeatus*), Tibetan catfish, and zebrafish.

The coding sequences obtained for single-copy gene families found in all 12 of these teleost species were aligned using MAFFT ( Yamada et al., 2016 ). Poorly aligned sequences were filtered using Gblocks ( Talavera and Castresana, 2007 ) and a maximum likelihood tree was then constructed using RAxML ( Stamatakis, 2014 ) with a GTRGAMMA substitution model and 1000 bootstrap replicates. Divergence time estimation was implemented using MCMCTree in PAML ( Yang, 2007 ) software, and three established fossil divergence times derived from the TimeTree database (http://www.timetree.org/) were used for calibration (Supplementary Table S13).

Gene families identified using OrthoMCL and divergence times estimated for *C. bouderius* and related species were used to assess the possible expansion and contraction of orthologous gene families using CAFÉ ( De Bie et al., 2006 ).

## Data Availability

The datasets presented in this study can be found in online repositories. The names of the repository/repositories and accession number(s) can be found in the article/Supplementary Material.
