# Supplementary material for: A chromosome-level genome of the helmet catfish (Cranoglanis bouderius)
Source: Front Genet. 2022 Aug 10;13:962406. doi: 10.3389/fgene.2022.962406 (PMC9400026; doi:10.3389/fgene.2022.962406)

***Supplementary materials***

**Supplementary Figures**

**Figure S1** Frequency distribution of the 17-mer graph analysis used to estimate the genome size of *Cranoglanis bouderius* (black line) and *K-mer* curve (blue dotted line) fitting was used to estimate the heterozygosity of *C. bouderius.*

**Figure S2** Distribution of gene length, CDS length, the numbers of exons and introns, and the length of exons and introns between *Cranoglanis bouderius* and related species.

**Figure S3** Image of *Cranoglanis bouderius* (Photographed by Yuan Xu).

**Figure S1**


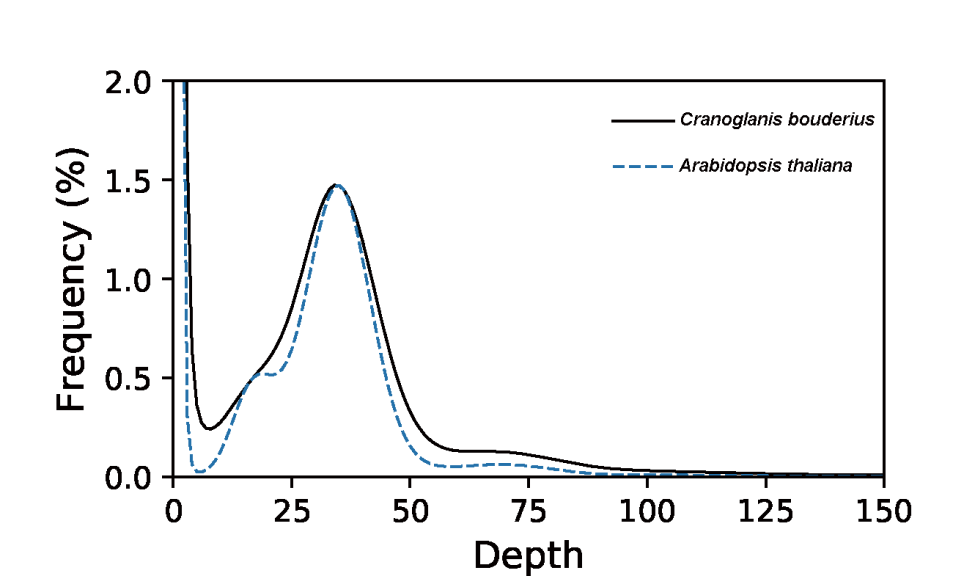


**
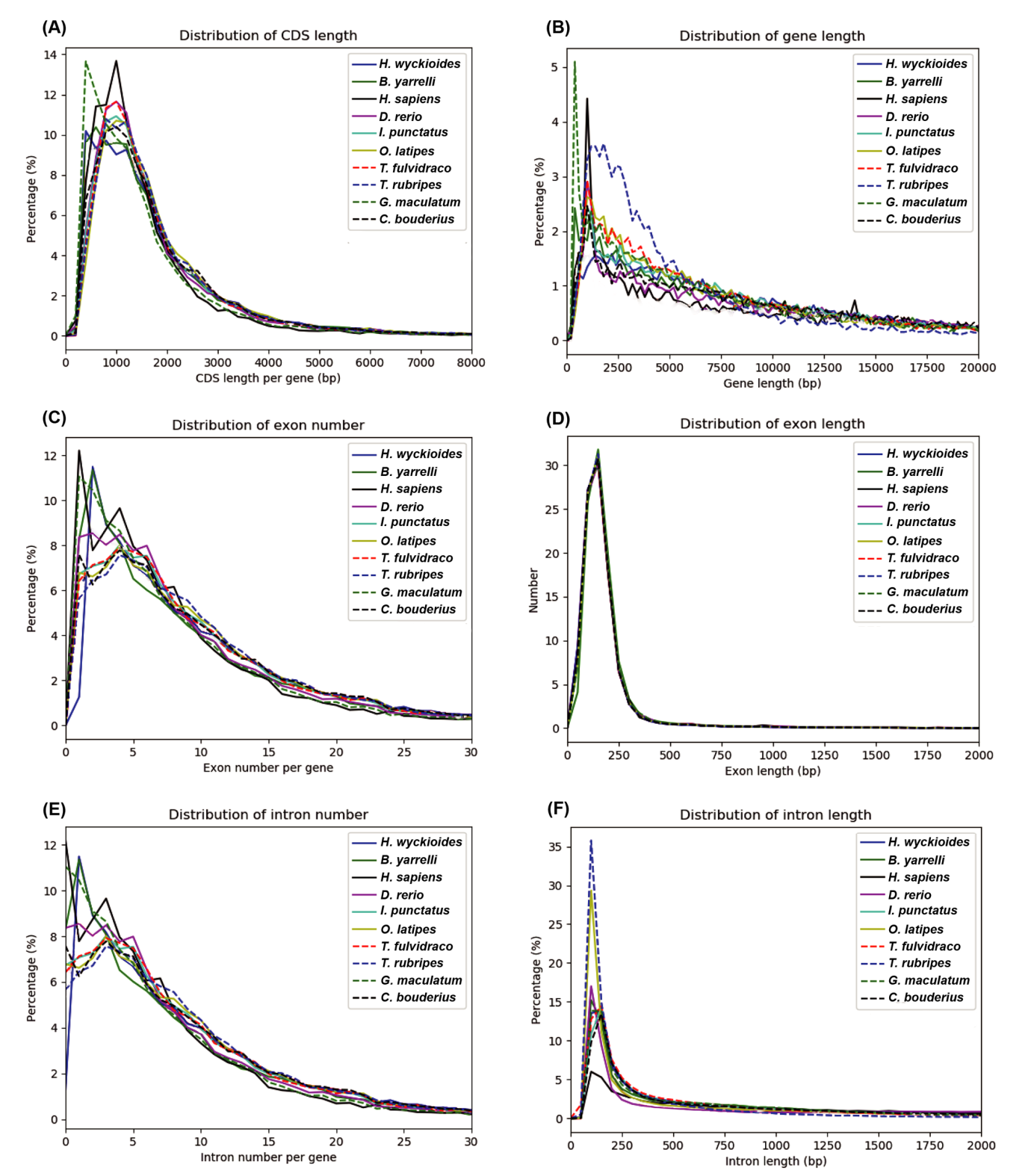
Figure S2**

**Figure S3**


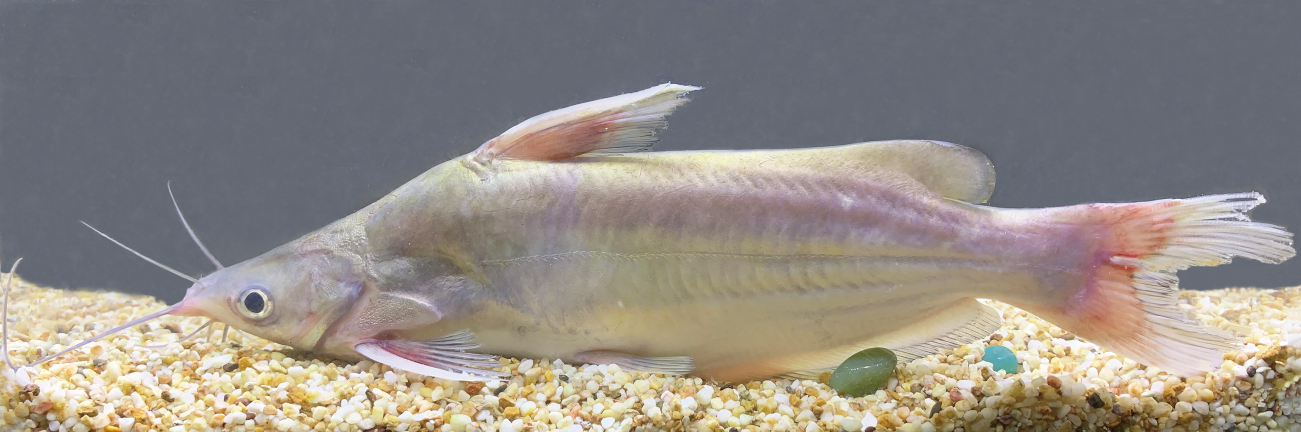

Supplement: Supplementary file 1 [file DataSheet1.docx]
